# A qualitative investigation of genetic counselors' experiences working with incarcerated patients

**DOI:** 10.1002/jgc4.70228

**Published:** 2026-06-06

**Authors:** Haley Fuoco, Elena R. Fisher, Rebekah Pratt, Krista Redlinger‐Grosse

**Affiliations:** ^1^ Department of Genetics, Cell Biology, and Development University of Minnesota Minneapolis Minnesota USA; ^2^ Department of Family Medicine and Community Health University of Minnesota Minneapolis Minnesota USA

**Keywords:** genetic counseling, incarcerated population, incarceration, inclusion

## Abstract

The United States has the highest rate of incarceration of any country and as the incarcerated population grows, health disparities increase. Genetic counseling provides essential healthcare that reduces health disparities through education regarding preventative screening and inherited health risks. Although genetic counselors (GCs) are involved in the care of incarcerated patients, there is limited research on the perspective of GCs working with this population. The purpose of this qualitative study was to explore the experiences of GCs working with incarcerated patients and explore opportunities to improve access and barriers to providing care. GCs who provided care for incarcerated patients were recruited in 2022 via the National Society of Genetic Counselors (NSGC). Ten interviewees from five different clinical specialties completed semi‐structured interviews that explored experiences working with incarcerated patients. Interviews were analyzed using reflexive thematic analysis, and four themes reflected participants' experiences: Prisons as the middle person; Scratching the surface of trauma; Perceptions of incarceration: Counselor bias; and Guidance and training needed. Participants' experiences described distinct differences in care for incarcerated patients given their navigation of the prison system and involvement of guards that changed the way they counseled, whether in the depth of providing psychosocial counseling and supports providing post‐test counseling. Participants had varied perceptions and emotional responses regarding care for incarcerated patients and thus sought guidance and training in working with this population. The participants' experiences illustrate how tenets of the Reciprocal‐Engagement Model of Genetic Counseling, specifically the importance of the counseling relationship, patient autonomy, and patient emotions in the genetic counseling process, are different in genetic counseling of the incarcerated population. Participants desired training on the needs of incarcerated patients for clinical practice and genetic counseling education. Study recommendations point to the need for formal genetic counseling guidelines for working alongside incarcerated patients.

**What is known about this topic**

Current literature examining genetic counseling in the incarcerated population highlights these services as a step toward health equity through: (1) a toolkit created in 2009 that describes considerations for GCs when providing services to this population, and (2) a perspective opinion piece that educates GCs on the unique needs of this population while promoting an abolitionist approach when providing care for this population.

**What this paper adds to the topic**

The present study takes the initial step in describing genetic counseling with incarcerated patients through a qualitative exploration of the experiences of genetic counselors working with incarcerated patients. Study results demonstrate that while there are similarities to general genetic counseling services, there are notable differences in providing genetic counseling services to incarcerated patients, including the involvement of guards, challenges in providing post‐test counseling, and emotional responses. As such, study recommendations point to the need for professional guidelines addressing how GCs can better support this patient population.

## 1 INTRODUCTION

The incarcerated population, which includes individuals in the custody of federal, state, and local correctional facilities, continues to grow yearly in the United States with over 1.8 million people incarcerated as of Spring of 2024 (Eber, 2009; Kang‐Brown & Zhang, 2024). As this population increases, needs are heightened for medical services to address the growing health disparities experienced by this population (Eber, 2009). GCs have the potential to play a role in providing accessible and accurate genetic risk information to this population, which has the potential to improve disparities in health outcomes. To date, however, research examining genetic counselors' perspectives on care for incarcerated patients is limited.

The right to medical care while incarcerated is required based on the United States Supreme Court's *Estelle v. Gamble* decision which concluded that the failure to provide health care while incarcerated falls under cruel and unusual punishment, a violation of the Eighth Amendment (Estelle v. Gamble, n.d.). Despite the Supreme Court ruling, incarcerated individuals often do not receive the appropriate healthcare, even when medically indicated or requested, due to systems placing heavy restrictions on access to health services (Dumont et al., 2021).

Lack of access to health services across several health specialties drastically impacts the physical and psychological well‐being of incarcerated individuals (Turney et al., 2012). Individuals from minoritized racial/ethnic populations are disproportionately impacted by health disparities as compared with non‐Hispanic Whites, and these disparities are further compounded by the intentional disproportionate rates of incarceration among these same communities (Eber, 2009; Interrupting Criminalization, 2023). Thus, individuals from racial/ethnic minorities are overrepresented in the prison system and underrepresented in access to adequate genetic services.

Oncology and reproductive genetic counseling services may be in the highest need for the incarcerated population. The prison population has higher cancer mortality than the general population; however, there is a lack of research on effective strategies to reduce cancer mortality in this population (Manz et al., 2021). The Federal Bureau of Prisons recommends the use of educational videos, group counseling, and health fairs to educate inmates on preventative health care related to cancer (Wilper et al., 2009). However, literature detailing the effectiveness and frequency of education on cancer prevention services for the incarcerated population is severely lacking.

Similarly, incarcerated pregnant individuals are more likely to experience poorer pregnancy outcomes due to the lack of prenatal care during incarceration, which can impact the health of the fetus and the pregnant person (Knight & Plugge, 2005). These disparities are notable given that nearly 1 in 20 incarcerated individuals are pregnant upon prison intake (Maruschak, 2008). To begin to address health disparities for incarcerated individuals, regular prenatal care and cancer prevention services should be routinely provided to this population (Sufrin et al., 2019).

GCs can play a crucial role in the healthcare needs of incarcerated individuals through preventative healthcare. GCs are uniquely trained to provide genetic risk assessment, facilitate decision‐making, and help patients understand their genetic health information (Barrett, 2022b; Resta, 2020). Therefore, GCs have the potential to reduce barriers for incarcerated individuals, especially given the field of genomic medicine's current work toward increasing access to services for underrepresented patient communities (Higgins, n.d.).

While existing literature highlights the importance of genetic counseling services in the incarcerated population as a step toward achieving health equity, limited publications describe genetic counseling practices with incarcerated individuals (Barrett, 2022a, 2022b; Miller, 2015; Ossege & Andrea, n.d.). A genetic counseling toolkit was published in 2009 to provide GCs with a training scenario involving an incarcerated pregnant person (Ossege & Andrea, n.d.). The objectives of the toolkit were to highlight the incarceration‐related health disparities of incarcerated patients, recognize factors linked to “criminal behavior”, outline laws and policies around medical services in prison systems, and describe strategies for building trust and eliciting medical histories for this vulnerable population (Ossege & Andrea, n.d.). The frequency with which this toolkit is utilized, and the genetic counseling outcomes are unknown. Additionally, aspects of the toolkit are notably outdated. For example, one learning objective detailing (“Factors linked to criminal behavior”) is now recognized as a stigmatizing approach given the multiple system‐level influences of oppression that contribute to disproportionate rates of incarceration in targeted communieies (Ossege & Andrea, n.d.; Wildeman & Wang, 2017).

More recently, a study was conducted on the importance of genetics education for incarcerated patients (Miller, 2015). This study, conducted at a prison where genetic counseling students provided genetics education in a classroom setting, highlighted the need for rapport building and trauma‐informed care when working with this population (Miller, 2015). Two opinion pieces, recently published in Perspectives, a genetic counseling commentary sponsored by National Society of Genetic Counselors (NSGC), illustrated how the genetic counseling population has historically disappeared incarcerated individuals (Barrett, 2022a, 2022b). The author, who had experience working with incarcerated populations, argued that GCs should adopt an abolitionist approach when working within this population, advocating for the end of incarceration as this is the only way to foster justice (Barrett, 2022a, 2022b). As such, discussions within the GC population have begun. Empiric and systematic knowledge around the experiences of genetic counseling with this population continues to remain sparse.

In summary, there is limited research describing GCs' interactions with incarcerated patients, as well as the impact of genetic services on health outcomes in this population. This study sought to begin to build this literature through the experiences of GCs working with the incarcerated population. This study is the first to identify the perspectives of GCs working with incarcerated patients to better understand how GCs can play a role in lowering health inequities.

## 2 METHODOLOGY

### 2.1 Positionality

We acknowledge our etic perspective to the field of genetic counseling, but emic perspective as individuals who have never been incarcerated and therefore, do not understand the experiences of incarcerated patients. HF, KRG, and ERF are genetic counselors with ERF having sole experience as a genetic counselor working with incarcerated patients. R.P. served as a qualitative expert in the study and has not had direct experience working with the incarcerated population. This is recognized to influence the data collection and analysis process given this provider perspective. A reformist framework has guided the entirety of this work.

### 2.2 Human subjects

The University of Minnesota Institutional Review Board (IRB) determined that this study did not meet the regulatory definition of research with human subjects and, therefore, considered exempt. IRB Exemption was procured on 09/15/2022.

### 2.3 Participants

The population of interest in this study included GCs in North America (i.e. United States or Canada) who were board‐certified or board‐eligible through American Board of Genetic Counseling (ABGC) who had participated in the clinical care of incarcerated patients and incarcerated parents of patients, for any clinical indication, within the last year. Potential participants were ineligible if they did not fully complete the online survey and/or were non‐English speaking.

### 2.4 Procedures

Study participants were recruited through the (NSGC) Student Research E‐blast (which was sent to approximately *N* = 4700 NSGC members) distributed initially on October 5th, 2022, and then a second E‐blast was distributed 2 weeks later (Appendix S1). The recruitment message described the study as research aimed at understanding the experiences of GCs who work with incarcerated patients (Appendix S1). No relationship with participants was established before the commencement of the study, and the first author introduced herself as a graduate student in genetic counseling with interests in genetic counseling with the incarcerated population.

At the end of the recruitment email, interested participants were provided with a link to a short, ~5–10 min, confidential online eligibility survey to screen for potential participants (Appendix S2). Specifically, the screening survey included questions pertaining to demographic information of the GC, whether the participant had clinical experience with incarcerated patients within the last year, information about the potential participant's state of residence and the type of healthcare institution they were employed by, and whether the participant would be interested in participating in a follow‐up semi‐structured interview via Zoom (Appendix S2). Eligible participants were sent an email invitation containing the rationale for the interview portion of the study, description of the study benefits and risks in an “Information Sheet for Research,” and logistics of interview scheduling (Appendix S3). Semi‐structured interviews were conducted by author H.F., a genetic counseling student, between October and December of 2022. Verbal consent was obtained from all participants at the beginning of the interview. Interviews were digitally recorded by Zoom software and stored in Box Secure Storage, which is a secure database for storing, sharing, and accessing sensitive, private, highly restricted files. Interviews were transcribed verbatim and audited by HF for accuracy and to remove all identifiable information from the final transcripts. The transcripts were not reviewed with or returned to participants.

### 2.5 Data collection tools

We developed the instruments for this study including a semi‐structured interview guide (Appendix S4). The semi‐structured interview guide included questions pertaining to appointment logistics, follow‐up care, education around participating in the care of incarcerated patients, and future recommendations for providing care for incarcerated patients (Appendix S4). Questions were developed based on existing literature and input from E.R.F given their previous experience working with incarcerated patients. The interview guide was piloted by one non‐participating GC identified by E.R.F. and K.R.G. as a GC with extensive clinical experience working with incarcerated patients in the prenatal setting, and minor revisions to the interview guide were subsequently made based on this feedback to include additional questions centered around logistical considerations for working with incarcerated patients (Appendix S4). This pilot interview was not included in the final dataset.

### 2.6 Data analysis

A constructivist approach was taken with the data using a reflexive thematic analysis process, a method to analyze qualitative data by identifying patterns of meaning in the data to capture themes (Braun & Clarke, 2006).

First, H.F. spent time becoming familiar with the data set by reading transcripts to understand each GCs experience working with incarcerated patients. After reading each transcript, H.F. reflected and organized reoccurring experiences noted by GCs. Then, H.F. manually coded the data to reflect similar phrases and words shared by GCs. Themes were developed through grouping similar codes identified by concept maps (Appendix S5). Coding was conducted iteratively and reflexively with transcripts continuously reviewed against emerging codes (Appendix S5). Once a concept map was generated to share the overall story of the data collected, themes were developed with written reflections also informing this process (Appendix S5). The research team (H.F., K.R.G, E.R.F., and R.P.) met to review throughout the analysis process to discuss coding and theme development (Appendix S5). Transcript data was analyzed using NVivo version 2.0 to facilitate the thematic analysis. The reporting of methodology follows the Reflexive Thematic Analysis Reporting Guidelines (RTARG; Braun & Clarke, 2024).

## 3 RESULTS

### 3.1 Participants

Ten GCs were interviewed and the majority self‐identified as non‐Hispanic White (90%), female (80%), and in the age range of 30–39 years old (70%). The two predominant practice areas for interviewees were the clinical specialties of cancer (30%) and prenatal/reproductive health (30%) with academic medical centers (80%) as the most common primary work setting. Most interviewees had counseled 1–5 incarcerated patients within the last year (70%), and work experience ranged from recent graduates (<1 year of experience) to counselors who graduated more than 10 years ago. Collectively, interviewees were licensed in 10 different states and represented a range of geographic regions. See Table 1 for additional interviewee demographics.

**TABLE 1:** Interviewee demographics.

Variable	Interviewees (*n* = 10)	
	*n*	%
Gender identity		
Female	8	80
Non‐binary	1	10
Prefer not to answer	1	10
Age range in years		
20–29	3	30
30–39	7	70
Race/ethnicity		
White	9	90
Prefer not to answer	1	10
Primary specialty area		
Cancer	3	30
Neurology	2	20
Ophthalmology	1	10
Pediatrics	1	10
Prenatal/reproductive health	3	30
Years of genetic counseling experience		
0–2	3	30
3–6	4	40
7–10	3	30
Type of clinical setting		
Academic medical center	8	80
Public, non‐profit health care system	2	20
Number of incarcerated patients within the past year		
1–5	7	70
6–10	2	20
11–15	1	10

### 3.2 Themes and sub‐themes

Four themes reflect the interviewees' experiences working with incarcerated patients: (a) prisons as the middle person; (b) scratching the surface of trauma; (c) perceptions of incarceration: counselor bias; (d) guidance and training needed.

#### 3.2.1 Prisons as the middle person

The interviewees stressed how genetic counseling of incarcerated patients always included working with prison systems. Prison systems were involved in all facets of the genetic counseling process, from the coordination of care, genetic counseling session with the presence of prison guards, and follow‐up communication.

The prison system determines if a genetic counseling visit can occur and when patients travel for medical visits. Some interviewees spoke about incarcerated patients' lack of autonomy in their choice to even attend the genetic counseling visit. For example, interviewees had experiences where patients expressed not wanting to come to a genetic counseling appointment because they recently lost family members and were not ready for genetic testing, but due to the system, they attended their appointment.

We have had a few sessions where the patient was forced to come to the appointment with us, even though they expressed that they did not want to come…her [the incarcerated patient] identical twin sister had just passed…they referred her because of the type of cancer he sister had, but the morning of the appointment she told me she was telling the guards she did not want to come…they said they would write her up if she refused to come to the appointment. (P‐5)

One of the consistent ways in which interviewees had direct contact with the prison system involved the prison guards' attendance during their appointments who always accompanied by patients to their genetic counseling appointment, although the number of guards depended on the prison institution.

The level of involvement of guards in the genetic counseling session varied:

I've had guards on their phones, listening loudly to YouTube videos and then I've had guards who are joking with the patient and interacting with the patient and then I've had guards who you forget they're in the room. (P‐3)

Some guards were disconnected from the session by sitting quietly or sleeping. Other guards interjected their own thoughts or questions into the session. Interviewees also described times when guards had strong relationships with patients and often joked around with them. There were times when guards made dehumanizing comments about the patient or caused harmful interjections. In these situations, interviewees were uncomfortable asking guards to leave because of rules requiring them to be present and were aware of the power dynamic of guards and how that impacted the patient but also impacted them.

Interviewees described barriers to providing support resources and providing referrals for incarcerated patients. Most interviewees stated they were unable to provide physical resources to incarcerated patients due to limitations from the prison system.

You couldn't give out resources to the patient. If there were any informational things, or even genetic testing results, you couldn't provide those. Just making sure to inform patients that any genetic test results would be released into their medical records, and that they could request their medical records through the hospital and then mail them to any family members. (P‐1)

After the initial session, most interviewees needed to follow up with incarcerated patients to discuss genetic testing results, provide support resources, and coordinate care. Interviewees noted barriers to providing direct and timely genetic testing results to incarcerated patients. Many described having to wait to disclose genetic testing results until the patient came back to the clinic for a follow‐up appointment; thus, patient preference for receipt of results via phone or an appointment was not available.

It's even difficult for me to try to get them [incarcerated patients] back to disclose results to them in person. I have one [result] that's over a year old that I've contacted the prison a couple of times to be like there's results, he needs to come in and talk to me, and I got nothing. (P‐8)

Others described the need to disclose genetic testing results to third party prison staff members with the hope the result would reach the patient. These experiences often left interviewees changing the way they discussed the result timeline to account for potential barriers.

The discussion of what happens next is much vaguer. Instead of saying, we're going to get these test results in 2–4 weeks, I will call you with this result. It's sort of like we will get these test results and we will let you know about them in the future. (P‐9)

Interviewees stated they often had to educate prison staff about genetic conditions and genetic testing results to make sure staff members could provide information to patients. Some interviewees stated challenges in their abilities to communicate with prison institutions which created barriers for patient care.

I feel like that whole process is quite different with an incarcerated person, and to explain the complexity having to do with an uncertain variance and the significance of that it's really impossible when you're working through multiple people who don't have a genetics background. In those cases, I have to simplify the information more so than I would like to and just say I am, or I'm not worried about this and just kind of leave it at the basic bottom line, so I do feel the results processes are definitely more difficult for these patients. (P‐2)

If any recommendations emerged from genetic testing results, it was often hard for interviewees to arrange follow‐up care for patients and their family members. If patients are incarcerated, their care and follow‐up is determined by prison system approval. If patients had a release date, it was often hard to coordinate care after release. Formerly incarcerated patients may move to a new area, have limited insurance coverage, and limited financial resources to proceed with follow‐up recommendations.

#### 3.2.2 Scratching the surface of trauma

Interviewees identified several instances where there were differences in the psychosocial skills utilized during genetic counseling sessions with incarcerated patients, and as a result, their genetic counseling often did not address the patient's deeper psychosocial and support needs. Interviewees had awareness about higher levels of trauma experienced by this population. Interviewees felt like they used less psychosocial skills due to concerns of bringing up trauma around incarceration or factors that lead their patients to become incarcerated.

It [the session] was less psychosocial focused than typical counseling would be, and I think that there is concern of like bringing up what led to the patient being incarcerated could open up some like bigger, more traumatic things that a GC isn't going to be meeting with them regularly to work through, and like being cognizant of that. (P‐1)

Interviewees expressed hesitations around addressing trauma without the ability to communicate with patients following the genetic counseling visit. Interviewees recognized barriers for incarcerated patients to receive mental health care. Therefore, they found it was unfair to incarcerated patients to discuss trauma without providing incarcerated patients with proper follow‐up resources.

Interviewees described avoiding certain topics or being aware of how certain topics may impact an incarcerated patient. They stated that often collecting a family history may be an area where trauma can surface with family dynamics and incarceration.

They [incarcerated patients] don't know anything about their relatives or what they've been up to, so keeping that in mind and being sensitive to that. (P‐2)

Incarcerated patients may have limited contact with their family members due to incarceration. Therefore, interviewees found this was an area of the genetic counseling session where they were more mindful about how they were asking questions and were limited in asking about sources of support that would typically come from family members.

#### 3.2.3 Perceptions of incarceration: Counselor bias

There was notable variation in the interviewees' opinions and perceptions on working with incarcerated patients. Some interviewees noted that they provided the same care regardless of incarceration status. They described not changing their perceptions or approach to the genetic counseling process based on incarceration status.

Although all interviewees know about a patient's incarceration status, they felt differently about the impact of the information. Some interviewees described concerns for their own safety and described changing their position in the room with an incarcerated patient, removing objects from the room, or turning around their name tags. In contrast, other interviewees were concerned about the safety of the incarcerated patients.

They [incarcerated patients] are really vulnerable, and I would say violence is something that I think about a lot, but I don't think about them being actors in [violence]. I think of them as being victims of violence more often than not. (P‐3)

There were differences in opinions regarding seeking out information regarding why an incarcerated patient is incarcerated with some using public records to learn about the reason for incarceration.

I don't know if this is necessarily something I should have done, but I did look up what they [incarcerated patient] were incarcerated for. It is public record, so you can search for it. My only concern was if it was something like a violent offense. I might remove certain objects from my desk. (P‐10)

Other interviewees felt strongly about not searching for the reason why a patient is incarcerated. They felt like it violated the patient's right to privacy.

No and we do not ask [about the reason for incarceration] I feel like I have a lot of discussions with students about that, and you know what would be the purpose of asking if it doesn't change anything. It shouldn't change how you interact with a patient. I sort of just put it out of my mind completely. (P‐9)

Interviewees discussed how they are impacted by discussions of trauma with incarcerated patients. Incarcerated patients may disclose information regarding previous traumatic events throughout their life. Some interviewees found that providing empathy for patients can be a helpful method in providing psychosocial support.

I read probably 5 or 6 of them [social work chart notes] when I had incarcerated patients and it was high trauma, vulnerable people who are making choices in desperation, who do not have a lot of access to options. I think of this a lot when I'm seeing these patients and lead with empathy. (P‐3)

Interviewees noted times where hearing about past trauma impacted their emotional responses. They felt like the patient's history was harder to empathize with as they could not imagine being in the patient's situation.

Overall, interviewees did utilize knowledge of incarceration status to inform their interactions with incarcerated patients. Some interviewees noted awareness about their behavioral changes, while others felt like they intentionally resisted behavioral changes.

#### 3.2.4 Guidance and training needed

Interviewees indicated the need for global guidance and training to aid in their work with incarcerated patients in the areas of: (a) practice, (b) training and education, (c) equity, and (d) research.

##### Practice

Interviewees advocated for established formal practice recommendations from NSGC for working with incarcerated patients and indicated that interviewees felt established recommendations would help GCs better advocate for genetic counseling services within prison systems. Guidelines would also help bring awareness to considerations for GCs working with this population.

I think that those type of practice guidelines, especially if they were written by a more formal body like NSGC or ABGC could be useful for us to point at to prisons to be like I know you have all these rules, but actually here are the standard guidelines, so we want to make a case that you need to change these rules to fit these guidelines of how we communicate with the patient. Should we be allowed a free subscription to their E‐messaging platform, those cost money. Should the patient be able to chat with us for free. (P‐6)

Interviewees also stressed the need for better follow‐up communication about test results and management recommendations with incarcerated patients. Some recommendations included access to calling the patient directly or building a system with the prison institution to help facilitate follow‐up communication.

##### Training and education

Interviewees received little formal incarcerated‐specific training in graduate school. Some training programs had opportunities for students to provide care to this population during clinical rotations or provide education around genetics in prison institutions, but most programs did not have these built‐in opportunities. At times, role plays were used as a means for genetic counseling students to learn more about psychosocial considerations for this population.

A big thing in our program was roleplays and doing practice. So, making sure to include role plays where someone is meeting with someone who might be incarcerated and giving students a chance to learn and think through what psychosocial concerns that come up. (P‐1)

##### Equity

Interviewees stressed the importance of equity in the field of genetic counseling and emphasized the need to provide the same care to every patient. One interviewee recommended using an abolitionist framework in dismantling the mass incarceration system. Other recommendations included ensuring incarcerated patients are valued and treated with autonomy and ensuring they are receiving follow‐up care, especially around mental health services.

I wish for greater access to medical and genetics care for these folks [incarcerated patients], and for greater access to mental health and other types of therapies and physical therapy and occupational therapy. (P‐2)

##### Research

Interviewees expressed frustration around the strict guidelines related to research for this population. Although guidelines were created to protect incarcerated individuals from harm, interviewees stated that these guidelines excluded incarcerated patients from equal opportunity to participate in clinical trials. Interviewees desired an incorporation of the voices of the incarcerated patients in conversations around guidelines impacting the healthcare they receive, as well as opportunities to participate in research.

GCs can also support further research in this area by participating in and conducting research projects that further the literature on genetic counseling with incarcerated patients.

Supporting research projects that are looking into this [topic] whether it be through encouraging more people to look into studies like this, and also to giving platform presentations on NSGC. And dedicating time to counselors talking about these barriers and access in the field as a whole. (P‐1)

## 4 DISCUSSION

This exploratory study is the first to describe GCs' experiences working with incarcerated patients. Results underscore the nuances and differences of providing care for incarcerated patients and recommendations for improving care through increased access and a reduction in barriers to genetic counseling services for this underserved patient population.

Study results noted important differences in the counselor–patient relationship when working with incarcerated patients. While the relationship remained central in their genetic counseling, participants utilized psychosocial skills differently and, in less depth, given the recognition of the unique psychosocial needs of incarcerated patients. It is important for GCs to acknowledge that incarceration itself is a source of trauma for patients (Morrison, 2023). Similar to Miller (2015), interviewees spoke to the need for genetic counseling skills around rapport building and trauma‐informed care when working with incarcerated patients (Miller, 2015). They noted the presence of past and present trauma and the impact on patients' trust in them as medical providers. Ultimately, this limited how they addressed psychosocial issues that they assessed or emerged during the session due to concerns about not being equipped to address deep‐seated trauma. This illustrates an ongoing tension within genetic counseling of the teaching vs. counseling model of practice and ways GCs conceptualize and manage their scope in addressing psychosocial issues (LeRoy et al., 2021) when working with underrepresented communities. Perhaps GCs are unconsciously leaning toward more of a teaching model of counseling, given an awareness of the depth of psychosocial needs in this population. It is important to consider, however, how this may be due to biases or discomfort with addressing the support needs of patients, especially given the extent of potential traumas experienced by this population. While, as is discussed below, GCs need to be sensitive to these traumas through more intentional practice of a counseling model steeped in a trauma‐informed perspective. It is important for GCs to acknowledge that incarceration itself is a source of trauma for patients. This points to the importance of bias awareness but also training on strategies to increase more trauma‐informed awareness and guided practice. A recent workshop on trauma‐informed practice for genetic counselors provided opportunities for strategies such as anticipatory guidance regarding appointment and subtly increasing autonomy by setting up rooms to decrease power imbalances (Schlub & De Deckker, 2025).

Participant experiences indicate that GCs and incarcerated patients are often left in the dark when it comes to control over the genetic counseling process. The Reciprocal‐Engagement Model (REM) of genetic counseling practice centers on the relationship of the patient and GC as central to the genetic counseling process and assumes that the patient and the GC mutually and equally participate in the genetic counseling process (McCarthy Veach, 2007). Mutual participation may be limited for GCs and incarcerated patients due to prison system rules and restrictions. There is an inherent power differential in any medical interaction, but this differential is intensified in the GCs' interactions with incarcerated patients. Examples of these limitations discussed by interviewees included incarcerated patients' shackles preventing them from physically engaging in the session, their inability to receive informational handouts, and lack of choice about coming to a genetic counseling appointment. The participation of guards at times was unsupportive and added another, obtrusive person into the genetic counseling room and thus, the relationship.

A novel finding of this study was the unspoken emotional influence of this work on GCs and their counseling process. Interviewees described a range of emotions around working with incarcerated patients, including anxiety and pride in their care. They also spoke about frustrations in working with the larger prison system and the constraints placed on them in patient follow‐up which sometimes meant not returning results promptly, if at all. Interviewees also acknowledged the undercurrent of trauma that was present in their care for incarcerated patients. Vicarious or secondary trauma can also be associated with compassion fatigue. It is important to be aware of the added toll placed on GCs (Braun & Clarke, 2006).

Additionally, it is important to note that GCs' biases (conscious or unconscious) toward working with incarcerated patients shape the assumptions and actions that may serve as barriers toward the GC–patient relationships. The need for self‐reflection and self‐care also was apparent in nuanced ways that GCs should be aware of conscious or unconscious perceptions about the population that led them to change their practice in potentially dehumanizing ways. An example of this was how GCs in this study indicated concerns around safety that influenced their perception of their patients. GCs held stigmatized beliefs around incarcerated patients that led them to change their behaviors toward their patients. The vast differences, even demographically, between genetic counselor workforce (e.g., race, gender, socio‐economic status) and the US incarcerated population creates an inherent power differential. This highlights the importance of self‐reflective practice to increase awareness of biases around incarceration and the impact that it has on a healthcare provider's view of incarcerated patients. As argued by Barrett (2022a, 2022b), the field of genetic counseling often forgets or does not include incarcerated patients in their advocacy work (Barrett, 2022a, 2022b). GCs have an opportunity and ethical obligation to advocate for their patients. NSGC Code of Ethics states “The counselor‐client relationship is based on values of care and respect for the client's autonomy, individuality, welfare, and freedom in clinical and research interactions” (Senter et al. 2018).

### 4.1 Recommendations

A range of considerations for working with incarcerated patients developed from interviewees in this study, as well as implications for GC practice, training and education, health disparities, and future research directions. Practical considerations and recommendations on providing care to incarcerated patients are illustrated in Table 2. These recommendations are noted to be from the perspectives of participants and providers in the data analysis and are not to be treated as evidence‐based recommendations. Additional reflection on how these recommendations may be informed by either a reformist vs. abolitionist perspective warrants examination and reflection. It is important to note that these recommendations may enforce or coexist with prison systems. Works by organizations such as Interrupting Criminalization can guide this reflection (Interrupting Criminalization, 2023).

**TABLE 2:** Recommendations for providing care to incarcerated patients.

Practice guidelines Prepare for the possibility of limited medical history Adjust expectations around family history information and ask sensitive questions about this topic Provide extra support in facilitating family communication around genetic testing results and follow up Adjust methods for building rapport that include increased time and sensitivity to potential lack of trust with medical system/providers Consult with clinic and prison system around managing the involvement of guards Recommend any necessary follow‐up care, like mental health counseling, with other providers or services Use trauma‐informed lens of care when assessing and addressing psychosocial concerns Seek self‐care and peer support with genetic counselors that have experience with this community
Equity Provide published clinical guidelines or recommendations on providing care for this community at the NSGC level Adopt an abolitionist approach around systems of mass incarceration Ensure incarcerated patients have autonomy over the genetic counseling process by communicating with the prison system to allow patients to have a choice in the process Increase access to genetic counseling in the incarcerated community by promoting education around the importance of genetic counseling to the incarcerated community and prison staff members Advocate for patients to receive genetic testing results directly from the genetic counselor Check biases around the incarcerated community to ensure practice aligns with humanism practices
Training and education Increase the number of roleplays or case examples involving incarcerated patients Include/increased education about the incarcerated community and genetic counseling service needs of this community Provide fieldwork training opportunities in settings that provide genetic counseling for incarcerated patients
Research Advocate for the perspectives of the incarcerated community in future genetic counseling research Support research projects aimed at identifying strategies to increase access to genetic counseling for this community
Suggested further reading “Abolition or Bust: Liberal Police Reform as an Engine of Carceral Violence” by Charlotte Rosen “Masked Racism: Reflections on Prison Industrial Complex” by Angela Davis “Police ‘Reforms’ You Should Always Oppose” by Mariame Kaba

Overall, interviewees noted there were no clear guidelines on working with incarcerated patients in a genetic counseling setting and called for practice recommendations to include strategies to manage the differences in genetic counseling care noted by study participants and highlight the main considerations for GCs to consider in working with incarcerated patients. Some GCs mentioned concrete guidelines through NSGC might help provide more guidance on working with this population.

As the field of genetic counseling is currently working toward increasing access to genetic counseling services through the Access to Genetic Counselor Services Act (Higgins, n.d.), incarcerated patients should be explicitly included in these efforts given they are an underserved population who experience health inequities (Wildeman & Wang, 2017). Results of this study also point to an increased need for training and education about working with incarcerated patients, whether through fieldwork experiences or didactic instruction. Bias awareness training should be required in this training given personal assumptions about the incarcerated populations, coupled with layers of power differentials, shape GCs' perspectives and unconscious actions and reactions. Suggested topics of reading are also included in Table 2 to provide greater historical context and understanding of mass incarceration. And finally, further research on the genetic counseling needs of the incarcerated population is needed, specifically research that involves the perspectives of incarcerated individuals and seeks to understand their perspective on the genetic counseling process.

### 4.2 Study limitations

While this is the first study to explicitly explore the experiences of GCs working with incarcerated patients in clinical settings, interviewees' experiences were based on recall of past experiences with incarcerated patients and were not direct observations of the sessions to characterize the appointment process and content more accurately. Participants in this study were limited to those recruited through the NSGC listserv, and thus, counselors who did not receive the NSGC listserv may have different or added perspectives.

Reformist vs. Abolitionist frameworks regarding the industrial prison complex (IPC) were not embedded within this study's design. We strongly encourage other GC researchers to consider how these IPC frameworks may impact the design and conduction of future studies and suggest having these discussions at the onset of study conceptualization. This may help to avoid de‐politicization of any resulting findings regarding genetic counseling interactions with incarcerated patients, which we recognize is a significant limitation of our exploratory study.

Most researchers in this study do not have firsthand GC experiences with incarcerated patients. This impacts the researchers' ability to fully understand GCs' experiences working with this population. The researchers were influenced by their personal experiences and values on this topic, which shaped the interpretation of results. The principles set by Braun and Clarke (2006) were utilized in the interpretation of the data (Schlub & De Deckker, 2025).

Incarcerated patients could not share their experiences with genetic counseling and thus, their perspective is missing in the description of the genetic counseling experiences and the conclusions of this study. Participant recommendations may not be reflective of incarcerated patients' recommendations about what is needed in genetic counseling to best support them in the genetic counseling process.

Due to the lack of professional guidelines, GCs are forced to resort to their own experiences and colleagues around them to determine best practices. This study provides a first glance into several important considerations advised by participants that may be considered in the absence of formal guidelines.

## 5 CONCLUSION

The present study provides a novel insight into exploring GCs' experiences in providing care for incarcerated patients. Study results highlight barriers and opportunities to improve access along with the perspectives of GCs providing care. Findings from this study support the need to increase education, training, and access in providing genetic counseling services to the incarcerated population.

## AUTHOR CONTRIBUTIONS

**Haley Fuoco:** Conceptualization (lead); data curation (lead); formal analysis (lead); funding acquisition (lead); investigation (lead); methodology (lead); project administration (lead); resources (equal); validation (equal); writing – original draft (lead); writing – review and editing (equal). **Elena R. Fisher:** Conceptualization (supporting); writing – original draft (equal); writing – review and editing (equal). **Rebekah Pratt:** Writing – review and editing (equal). **Krista Redlinger‐Grosse:** Conceptualization (equal); data curation (equal); formal analysis (equal); funding acquisition (supporting); investigation (supporting); methodology (equal); resources (equal); validation (equal); writing – original draft (equal); writing – review and editing (equal).

## CONFLICT OF INTEREST STATEMENT

Haley Fuoco, Krista Redlinger‐Grosse, PhD, ScM, CGC, Elena R. Fisher, MS, CGC, and Rebekah Pratt, PhD declare that they have no conflicts of interest.

## ETHICS STATEMENT

Human studies and informed consent: All procedures followed were in accordance with the ethical standards of the responsible committee on human experimentation (institutional and national) and with the Helsinki Declaration of 1975, as revised in 2000 (5). Informed consent was obtained from all participants for being included in the study.

Animal studies: No animal studies were carried out by the authors for this article.

## Supporting information


Appendix S1

Appendix S2

Appendix S3

Appendix S4

Appendix S5

## Data Availability

Data for this study are available upon request from the corresponding author. All data obtained during this study that are relevant to the major research questions are reported in the published article, tables, and Supplementary material.
