# Supplementary material for: A qualitative investigation of genetic counselors' experiences working with incarcerated patients
Source: J Genet Couns. 2026 Jun 6;35(3):e70228. doi: 10.1002/jgc4.70228 (PMC13241912; doi:10.1002/jgc4.70228)
Supplement: Supplementary file 1 — Appendix S1 [file JGC4-35-0-s001.pdf]

Appendix S1: Recruitment Email

Do you have experience working with incarcerated patients? We want to hear from you!

We are conducting a research study aimed at understanding the experiences of genetic counselors working with incarcerated patients to better understand differences in patient care for this population. If you are interested in learning more about this research and the potential to participate in a follow-up Zoom interview, please click the link below to take a screening questionnaire.

All genetic counselors practicing in the United States or Canada who have experience working with incarcerated patients are eligible to take this survey.

*Please contact Haley Fuoco (*fuoco002@umn.edu*) or my research advisor, Dr. Krista Redlinger-Grosse (redli009@umn.edu) with any questions or concerns.*

[SURVEY LINK]

Thank you for your time and consideration.

Sincerely,

Haley Fuoco

Genetic Counseling Graduate Student

University of Minnesota
