# Supplementary material for: A qualitative investigation of genetic counselors' experiences working with incarcerated patients
Source: J Genet Couns. 2026 Jun 6;35(3):e70228. doi: 10.1002/jgc4.70228 (PMC13241912; doi:10.1002/jgc4.70228)
Supplement: Supplementary file 2 — Appendix S2 [file JGC4-35-0-s002.pdf]

Appendix S2: Screening Survey

Thank you for your interest in this research study! The following questions will help us understand the amount of experience you have working with incarcerated patients and to ask your interest in participating in a more in-depth semi-structured interview.

This survey should take approximately 5-10 minutes to complete. You are free to decline to answer any and all questions for any reason, at any point during this survey.

1. Have you been involved in the clinical care of an incarcerated patient, including coordination of care or full genetic counseling, within the last year?
    a. Yes
    b. No

If the participant answers no, skip to the end of the survey.
If the participant answers yes, continue with the survey

[Practice Based Questions]
1. How many incarcerated patients have you counseled or been involved in the clinical care of within the past year?
    - 1-5
    - 6-10
    - 11-15
    - 15+
2. What specialty of genetic counseling have you seen most of your incarcerated patients within?
    - Prenatal/Reproductive Health
    - Infertility/IVF
    - Cancer
    - Pediatrics or General Genetics
    - Cardiology
    - Neurology
    - Other
        - Please specify
3. How many years of experience do you have as a genetic counselor?
    - Numerical response
4. What state(s) or province(s) do you currently practice in?
    - Drop-down option with all US States and territories along with Canadian provinces.
5. What type of clinical setting do you practice in?
    - Public, non-profit healthcare system
    - Private, non-profit healthcare system
    - Academic medical center

- Private clinic or company

6. Is there anything important you think we should know about your work with incarcerated patients? (short answer)

[Basic Demographics]

1. What is your age?
   - Numerical free response

2. What race do you identify as?
   - American Indian or Alaska Native
   - Asian
   - Black or African American
   - Latine
   - Multi-racial
   - Native Hawaiian/Pacific Islander
   - White
   - Other
     - Please specify
   - Prefer not to answer

3. What ethnicity do you identify as?
   - Hispanic
   - Non-Hispanic
   - Prefer not to answer

4. What is your gender identity?
   - Female
   - Male
   - Non-binary
   - Transgender female
   - Transgender male
   - Other
   - Please specify
   - Prefer not to answer

1. Are you willing to participate in a 30-45 minute Zoom interview?
   - Yes
     - Phone number:
     - Email:
   - No

Thank you for taking the time to complete this survey. If you are selected to participate in this study, you will be contacted for a phone interview via email.
