# Supplementary material for: A qualitative investigation of genetic counselors' experiences working with incarcerated patients
Source: J Genet Couns. 2026 Jun 6;35(3):e70228. doi: 10.1002/jgc4.70228 (PMC13241912; doi:10.1002/jgc4.70228)
Supplement: Supplementary file 3 — Appendix S3 [file JGC4-35-0-s004.pdf]

Appendix S3: Information Sheet for Research

INFORMATION SHEET FOR RESEARCH Genetic Counselors' Experiences Working with Incarcerated Populations

You are invited to be in a research study of the genetic counseling experience working with incarcerated patients. You were selected as a possible participant because of your survey response. We ask that you read this form and ask any questions you may have before agreeing to be in the study. This study is being conducted by: Krista Redlinger-Grosse, Genetics, Cell Biology, and Development Procedures: If you agree to be in this study, we would ask you to do the following things: Complete a 45 minute semi-structured interview via Zoom Confidentiality: During the project, information from this study will be kept private and will be stored securely. Only the research team will have access to information that identifies you. Your identifying information will not be shared with others outside of this research study. However, organizations that may inspect and copy your information include the Institutional Review Board (IRB), the committee that provides ethical and regulatory oversight of research, and other representatives of this institution, including those that have responsibilities for monitoring or ensuring compliance (such as the Quality Assurance Program of the Human Research Protection Program (HRPP)). Any personal information that could identify you will be removed or changed before we publish any report or share the results or data from this study. Voluntary Nature of the Study: Participation in this study is voluntary. Your decision whether or not to participate will not affect your current or future relations with the University of Minnesota [or, if with other cooperating institutions, insert names here]. If you decide to participate, you are free to not answer any question or withdraw at any time without affecting those relationships. Contacts and Questions: The researcher(s) conducting this study is (are): Haley Fuoco and Krista Redlinger-Grosse. You

may ask any questions you have now. If you have questions later, you are encouraged to contact them at University of Minnesota, Genetic Counseling Program, 224-531-8357, fuoco002@umn.edu. Advisor: Krista Redlinger-Grosse, University of Minnesota Genetic Counseling Program, 612-626-1900, redli009@umn.edu This research has been reviewed and approved by an IRB within the Human Research Protections Program (HRPP). To share feedback privately with the HRPP about your research experience, call the Research Participants' Advocate Line at 612-625-1650 (Toll Free: 1-888-224-8636) or go to z.umn.edu/participants. You are encouraged to contact the HRPP if: HRP-587 Template Version: 06/30/2022

● Your questions, concerns, or complaints are not being answered by the research team.

● You cannot reach the research team.

● You want to talk to someone besides the research team.

● You have questions about your rights as a research participant.

● You want to get information or provide input about this research. You will be given a copy of this information to keep for your records.
