# Supplementary material for: A qualitative investigation of genetic counselors' experiences working with incarcerated patients
Source: J Genet Couns. 2026 Jun 6;35(3):e70228. doi: 10.1002/jgc4.70228 (PMC13241912; doi:10.1002/jgc4.70228)
Supplement: Supplementary file 4 — Appendix S4 [file JGC4-35-0-s003.pdf]

Appendix S4: Interview Guide

Opening Statement: Hello and thank you for responding and taking the time to talk with me today. I'm Haley Fuoco. I am a second-year graduate student in the University of Minnesota Genetic Counseling Program. This interview is part of my master's scholarly project where I am hoping to better understand the experiences of genetic counselors working with incarcerated patients. I want to remind you that this interview is entirely voluntary, and you can choose not to answer any and all of the questions with no consequences. I will also be recording the interview so I have your responses available as we continue with this study, but any of your responses included in the results will be anonymized. All this being said, are you comfortable continuing with this interview?

Questions:

1. What is your specialty, type of healthcare system/employer, and general types of referrals or patients you see.
   - Are they the primary health system in their area that sees most incarcerated patients?
   - Do they generally get referrals from all over or is this more of a private health system where they don't see Medicaid patients?
2. What indications do these patients usually come to the clinic for?
   - What channel do these patients use to have an appointment with you?
3. I am going to ask a few questions to understand if there are differences in how sessions are tailored for incarcerated patients:
   - Do you allow more or less time for the appointment?
   - Does your chart review or preparation for the appointment differ at all, if so how?
   - Do you anticipate more or less additional clinical or social needs for an incarcerated individual? If so, do you follow up about these needs any differently than nonincarcerated patients?
4. Does your physical location or the tools you might use (ex. Visual aids) differ at all for incarcerated individuals?
5. Do you provide more or less information about genetic counseling content and/or general clinical care (such as next appointment, other testing options, etc.) for incarcerated patients?
6. If a referral needs to be made (ex: mental health resources), does your process differ or how do you approach follow up needs any differently?
7. Can you describe any unique aspects of the flow of clinical care including the referral process, being seen in the clinic, coordinating testing, and follow up?
   - What are patients' understanding of the appointment?
   - Does it seem like patients are informed about the utility/reason for genetic counseling compared to nonincarcerated patients.
   - What has your experience been with follow up care?
8. How did you learn this information? (insurance, follow up appointments, communication with the prison system, etc.)

9. Can you tell about the logistics of appointments involving incarcerated patients and your perceptions of how differences in these appointments compared to nonincarcerated patients might impact patients.
    - How do differences in these appointments impact a patient's psychosocial state, ability to concentrate, testing decisions, rapport building?
10. Do you have any recommendations and/or things you wished were different for caring for this community in the future?
    - On a general counseling level or on a system level
11. Anything you think would be important to add that I haven't already asked about today?

Closing Statement: That is all the questions I have for you today. Thank you so much, again, for participating in this interview and sharing some of your experiences with me. I am no longer recording these conversations. If you have any questions about this project, please feel free to contact me or any of the other contacts listed on the interview invitation.
