# Supplementary material for: A qualitative investigation of genetic counselors' experiences working with incarcerated patients
Source: J Genet Couns. 2026 Jun 6;35(3):e70228. doi: 10.1002/jgc4.70228 (PMC13241912; doi:10.1002/jgc4.70228)
Supplement: Supplementary file 5 — Appendix S5 [file JGC4-35-0-s005.pdf]

Appendix S5: Codebook

Theme & Subthemes	Description
**Perceptions of Incarceration: Counselor Bias**	• GCs noted stigma around incarceration status<br>• Looking up vs. not looking up a patient:<br>-invasive<br>-patient privacy<br>-concerns for safety<br>• Intentionally making aware or not aware of incarceration status<br>• Unable to be "put in their shoes"<br>• Provide the "same care"<br>• Intimidated by the presence of shackles and guards
**Scratching the Surface of Trauma**	• Unable to address trauma<br>• Fear of unlocking or surfacing trauma<br>• Lack of bandwidth to address psychosocial concerns for this community<br>• Limited mental health support for follow-up<br>• Being mindful of what is said during the visit<br>• Emotionally draining
**Prisons as the Middle Person**	• Guards present and involve for visits (varying level of participation in visits)<br>• Prison determines approval for visits and genetic testing<br>• Prisons determine if results are disclosed to patients<br>• Determine what materials patients are allowed to receive from GCs<br>• No control over when patients return for results disclosure<br>• Cannot communicate with patients directly<br>• If the patient is released, barriers to accessing follow-up care due to

	limited resources and support, along with moving to a different area
**Guidance and Training Needed**	• Recommendations shared by interviewees for future care for this community<br>• Recommendations around the genetic counseling process<br>• Recommendations around genetic counseling training and continued education on the incarcerated community<br>• Recommendations around increasing equity and access for the incarcerated community.<br>• Recommendations around increasing research around the incarcerated community in the field of genetic counseling
